# Prediction of Attention Groups and Big Five Personality Traits from Gaze Features Collected from an Outlier Search Game

**DOI:** 10.3390/jimaging10100255

**Published:** 2024-10-16

**Authors:** Rachid Rhyad Saboundji, Kinga Bettina Faragó, Violetta Firyaridi

**Affiliations:** 1 Department of Artificial Intelligence, Faculty of Informatics, ELTE Eötvös Loránd University, Pázmány Péter Sétány 1/A, H-1117 Budapest, Hungary; 2 Nokia Bell Labs, Bókay János u. 36-42, H-1083 Budapest, Hungary; violettaff@gmail.com

**Keywords:** eye tracking, gaze-based interaction, virtual reality game, visual attention, personality traits

## Abstract

This study explores the intersection of personality, attention and task performance in traditional 2D and immersive virtual reality (VR) environments. A visual search task was developed that required participants to find anomalous images embedded in normal background images in 3D space. Experiments were conducted with 30 subjects who performed the task in 2D and VR environments while their eye movements were tracked. Following an exploratory correlation analysis, we applied machine learning techniques to investigate the predictive power of gaze features on human data derived from different data collection methods. Our proposed methodology consists of a pipeline of steps for extracting fixation and saccade features from raw gaze data and training machine learning models to classify the Big Five personality traits and attention-related processing speed/accuracy levels computed from the Group Bourdon test. The models achieved above-chance predictive performance in both 2D and VR settings despite visually complex 3D stimuli. We also explored further relationships between task performance, personality traits and attention characteristics.

## 1. Introduction

The field of human–computer interaction (HCI) has evolved by integrating insights from cognitive and behavioral sciences to enhance user experiences. One key aspect is personality, the patterns of thoughts, feelings, and actions that make us unique, as defined in the five-factor model: extraversion, neuroticism, open-mindedness, agreeableness, and conscientiousness [1].

Scientific studies show that personality significantly impacts how individuals interact with systems and interfaces [2,3,4]. Therefore, it can be claimed that personality-based interface design serves various domains and applications [5].

In parallel to personality, attention—another key cognitive function—holds an important role within the HCI context. Individual variations in attentional allocation during engagement with interfaces critically influence both user experience as well as task performance [6]. Traditional tools like the Group Bourdon test [7] have been utilized to measure this sustained attention [8]. Difficulties sustaining attention can affect maintaining stable performance on prolonged tasks, causing fatigue [9]. Moreover, research shows that attentional capacities fluctuate, occasionally causing attention lapses that result in undesired actions, even in optimal conditions [10].

To gain insights into these cognitive and behavioral processes, researchers have turned to the study of eye movements. Eye tracking, as a way to measure eye movements, provides insights into visual attention, behavior, emotions, and other cognitive processes [11]. Using machine learning approaches, features based on eye movements have proven effective in predicting various aspects of human behavior, such as visual attention [12,13], problem-solving [14,15], cognitive load [16,17], and personality traits [18,19,20].

Building upon research on cognition, behavior, eye tracking and virtual reality (VR) technologies provides a platform for exploring these mechanisms in realistic yet controlled settings. In the past decade, VR has initiated a paradigm shift across various sectors, including education, professional training, and business [21,22,23]. By integrating eye tracking, VR technologies allow for an in-depth study of behaviors, attention, and task performance, providing objective gaze data on individual differences.

The capability of such novel interaction via multimodal data processing is only one part of the truly effective cooperation between humans and computers. The ability to adapt to individual human needs is key to increasing the effectiveness and acceptance of these systems [24]. Rather than looking at complex, multimodal manufacturing systems, we focused on readily available, off-the-shelf tools for multimodal data capture and associated methodologies. In our experiments, we take advantage of an immersive VR environment combined with eye tracking to provide users with one of the least intrusive but most enjoyable data capture environments.

Previous research in personality prediction using gaze data has largely been conducted in simplistic 2D stimuli on desktop displays [2,3,4,25,26], which, while providing valuable insights, have limited the ability to replicate the complexity and immersion of real-world scenarios. One of the few exceptions is the study by Hoppe et al. [18], which measured eye movements in everyday scenarios to predict personality traits, demonstrating the potential for more naturalistic environments. Nevertheless, this study has drawbacks due to the complexities and unpredictability of real-world contexts. Consequently, these 2D studies may still not capture complex real-world cognitive behaviors and contexts to the same extent as immersive VR environments [27,28].

Predictive modeling specifically using the Group Bourdon test as an attention measure has not yet been fully exploited in 2D or VR environments. Simultaneously, the potential of using gaze features to predict personality traits in the context of a 3D stimulus game within these environments has not been fully explored.

**Figure 1:**
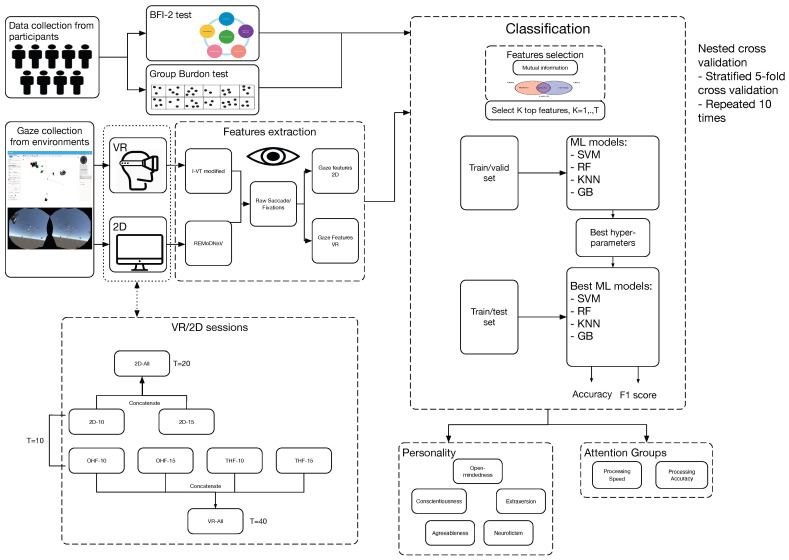
Schematic representation of our proposed classification pipeline. The process starts with the collection of personal data and the recording of game logs. We then extract gaze features from both 2D and VR data sets. In the VR/2D sessions’ box (see (**lower left corner**)), T refers to the total number of features. As the next step of the pipeline, we apply feature selection within nested cross-validation to classify personality traits and attention groups.

Figure 1 presents a visual overview of our proposed classification pipeline. In this scientific work, we provide the following major contributions:

1. We designed and developed a classification pipeline that incorporates machine learning models to predict self-reported personality traits (extracted from the BFI-2 test [29]) and attention-related characteristics (derived from the Group Burdon test) from gaze data.
2. We investigated the influence of personality traits and attention-related attributes on participants’ task performance in an outlier search game.
3. We studied the effects of factors such as hand control type, task duration, and game environment (2D vs. VR) on the performance of the classification models.

The main outcome of our work aims to address the basic limitations of the adaptive interface research domain by developing models that can predict attentional attributes from gaze data, as well as classify personality traits and assess these traits and attention levels’ impact on task performance. The further objective of our research is to explore the feasibility of a user-centred interface design through easily implementable data collection methods.

The rest of this paper is organized as follows. Section 2 overviews the related theoretical background and other relevant scientific works that highlight the visual search, eye movement, human attention and personality in general and are linked with virtual reality topics. Section 3 introduces the developed outlier search game, the experimental design, collected and derived data, and data pre-processing details together with feature extraction and training of the classification models. Results with numerical visualizations are shown in Section 4. Section 5 includes a discussion of the findings, with separate sections on correlation analysis and classification outcomes. Finally, Section 6 and Section 7 discuss the limitations and conclude our work.

## 2. Related Work

The background literature relevant to this work spans several key domains, including visual search behavior, personality and attention assessment in traditional settings, and the use of virtual reality technologies to study these psychological constructs.

In the following subsections, we first review research on visual search and performance, with a focus on findings related to individual differences. Next, we survey important work investigating associations between eye movement, personality traits as well as attention using traditional experimental paradigms. We then examine recent studies centered on virtual reality systems for analyzing personality expression and attention mechanisms in immersive environments.

### 2.1. Visual Search

Visual search, the perceptual process of selectively guiding gaze to find targets in a scene, involves complex interactions between bottom-up salience, top-down guidance, scene structure, search history, and target value, as described by Wolfe and Horowitz [30]. Moreover, beyond these external drivers, the role of individual differences is increasingly recognized to influence visual search performance. For example, Peltier [31] found that individual characteristics like working memory capacity, vigilance, and attentional control can predict performance in low-prevalence visual search tasks.

Specifically, the relationship between personality traits and visual search has been a focus of recent research. Conscientiousness, one of the Big Five personality dimensions, was found to correlate positively with visual search accuracy by Biggs et al. [32]. Additionally, Woods et al. [33] demonstrated visual preferences could predict personality traits. Furthermore, the effects of personality extend to other individual factors as well, with research indicating sex-specific personality traits [34] and Autism Quotient scores [35] influence visual search strategies.

This indicates that visual search guidance integrates external attentional drivers with person-specific internal factors that interact to influence gaze patterns and attentional allocation. Additional characterization of these individual mediators could enable the development of intelligent search systems capable of dynamically predicting and enhancing visual hunting performance in an individually tailored manner.

### 2.2. Eye Movements, Attention and Personality

This subsection provides an insight into the recent scientific literature based on gaze data, attentional mechanisms and personality traits used in different research scenarios.

Research on gaze data has largely focused on predicting cognitive and emotional states. Skaramagkas et al.’s study [36] presents an example of how gaze-related features can be effectively employed to predict emotional arousal and valence in emotionally charged situations. Appel et al. [17] demonstrated how eye movements can be used to infer cognitive load. Similarly, Jaques et al. [37] investigated educational settings and explored the predictive capability of gaze data in detecting student disengagement in intelligent tutoring systems. Finally, Zhou et al. [38] expanded this cognitive and emotional predictive perspective to a practical setting by forecasting situational awareness in automated driving. These studies suggest that combining gaze with context-specific information can enhance state prediction.

A secondary area of research revolves around the use of gaze data for the enhancement of task performance. Raptis et al. [39] demonstrated how gaze data can expose cognitive strategies in pattern recognition tasks. Huang et al. [40] utilized gaze patterns to predict task intent in collaborative interactions. These studies suggest that analyzing gaze patterns can expose cognitive strategies, which could allow for the tailored design of systems that optimize performance in diverse tasks and collaborative settings. In addition, gaze data have proven useful not only for predicting cognitive and emotional states but also for examining individual differences and predicting human errors. Dumais et al. [41] emphasized the relevance of individual differences in gaze patterns during web searches. Following this, Kasneci et al. [42] harnessed gaze data to discern individual differences in IQ tests, indicating the potential for gaze data to reveal cognitive variability. Lastly, Saboundji et al. [43] leveraged both gaze and cursor movements to predict human errors in a divided attention task.

Recent studies have demonstrated the potential for using gaze patterns as predictive biomarkers for personality traits. Leveraging eye tracking technology to unobtrusively measure visual attention provides unique insights into the links between gaze behavior and individual differences in personality. The pivotal study of Hoppe et al. [18] first showed that eye movement patterns during everyday tasks could effectively predict four of the Big Five traits—neuroticism, extraversion, agreeableness, and conscientiousness. Chen et al. [44] examined gaze behavior during interactions with recommendation interfaces, connecting personality to user preferences. Berkovsky et al. [19] then introduced new frameworks using multimodal eye tracking and physiological data during image and video viewing to objectively infer traits. While controlled lab studies have provided a valuable starting point, future research must address potential biases and limitations by moving towards more naturalistic VR and real-world settings. Overall, innovative methodologies leveraging gaze data show promise for reliably assessing personality but require further validation outside controlled environments.

In parallel, the prediction of attention behaviors has seen increasing progress due to the application of machine learning methods. Initial explorations, such as the work of Van Der Linden et al. [9], linked professional burnout to attentional difficulties, signifying cognitive deficits in stressed individuals. Notably, in the pursuit of enhanced precision and real-world applicability, attention prediction has increasingly incorporated gaze and other multi-modal data. Li et al. [45] employed a multimodal approach to detect human attention in e-learning using facial expressions, gaze, and mouse dynamics. The potential of gaze information has been further illuminated by Broussard et al. [46] and Zaletelj et al. [47], who used VR and Kinect-based systems in classroom scenarios, respectively, to enhance the teaching–learning experience through attention-aware interfaces.

Despite these strides, a more robust understanding of the intersection between gaze data and attention mechanisms remains to be fully explored. For instance, the recent works of Hassan et al. [48] and Xu et al. [49] demonstrated compelling advancements using EEG signals and multi-level attention recognition methodologies, but the role of gaze data was not specifically addressed. Similarly, attention prediction studies by Shavit-Cohen et al. [50] and Singh et al. [51] utilized virtual reality and deep neural networks, revealing complex attention dynamics but not explicitly involving gaze. It can be argued that combining gaze tracking with robust attention measures represents a promising approach to gaining deeper insights. However, establishing ground truth remains difficult, as attention has varied definitions and measurement methods. This complexity hinders the development of consistent predictive algorithms.

### 2.3. Virtual Reality and Personality

The study and analysis of human behavior and personality in VR is a broad research topic. Here, we summarize relevant studies related to our paper.

The rapid evolution of virtual reality (VR) technology in recent years has opened new frontiers for studying the expression and measurement of personality in immersive virtual environments [52]. While personality assessment has traditionally relied on self-reported questionnaires prone to biases and real-world observations limited in experimental control, VR provides a medium to systematically simulate realistic situations and analyze how traits manifest through embodied interactions and responses [53].

Bouchard et al. [54] pioneered the use of VR in studying the impact of personality traits on fear responses in phobic environments. Subsequent work by Slater et al. [55] demonstrated the influence of VR on real-world attitudes and behaviors, showing that users’ identification with their avatars could have significant real-life implications. VR studies provide external validity to classic personality frameworks, affirming that our deeply ingrained traits and dispositions shape social dynamics, emotional processing, and subjective perceptions alike in both physical and digitally simulated worlds. This narrative was further enriched by the work of Bailey et al. [56], who demonstrated that traits such as extroversion and neuroticism could predict user behavior within VR.

A parallel body of research is exploring how individual differences impact performance within virtual reality environments. While some studies have investigated the relationship between personality traits and VR performance, the findings have been inconclusive. Rosenthal et al. [57] found that although surgical residents exhibited personality traits different from the general population, these distinct traits were not predictive of technical performance in virtual reality laparoscopic tasks. However, more recent research has identified specific personality traits that correlate with improved performance in VR. Katifori et al. [58] found that specific personality traits strongly correlated with better task performance in a virtual reality environment involving object manipulation. These results suggest that further research is needed to more fully understand how personality impacts VR interaction and performance.

Moreover, researchers are leveraging machine learning methods to gain insights from human behavior in virtual reality. Parra et al. [59] used machine learning by combining eye tracking and behavioral data to classify individuals based on leadership style in a virtual workplace. They found that eye tracking measures contributed strongly to discrimination of leadership styles. Along these lines, Gao and Kasneci [60] showed that eye tracking data alone can predict users’ experiences with different VR locomotion techniques, revealing subconscious responses. Khatri et al. [61] similarly demonstrated that a combination of eye tracking, posture, and interaction data could detect users’ personality traits during tasks in a virtual store. Moreover, Gao et al. [62] extended such insights to the educational field by investigating gender differences in computational thinking skills using students’ eye movements in an immersive VR classroom. Their models achieved over 70% accuracy in gender classification, indicating that eye tracking features can provide discriminative information in educational contexts as well. By analyzing eye movements and other unconscious responses to VR stimuli, researchers were able to predict leadership style, user experience, personality traits, and cognitive abilities, revealing new insights into how individuals interact with and perceive virtual worlds. As these techniques advance, they have the potential to enhance the design of personalized VR systems that optimize experiences for users’ specific traits, needs, and contexts.

### 2.4. Virtual Reality and Attention

Research underscores attention’s vital role in VR experiences, as demonstrated across key studies. Bowman et al. [21] established that immersion alone does not determine VR outcomes—interactive components are critical for engaging attention. Building on this, Bouchard et al. [54] revealed that heightened subjective presence and anxiety arise in emotionally provocative VR settings compared to neutral environments. This suggests VR scenarios may influence attention by eliciting arousal. Furthermore, Seo et al. [63] showed that virtual avatars in educational VR enhanced attentional processing versus VR alone. In a practical context, Mosteanu [64] determined multimedia techniques best maintain student attention during remote VR lessons, pointing to the fragility of attention without varied stimuli. Moreover, Prpa et al. [65] used respiration-linked audiovisual feedback to elicit and sustain focused breath awareness during VR meditation. Together, these studies highlight the vital but multifaceted role of attention in determining the outcomes of different virtual reality applications. In addition, they identify the need for further investigation of individual differences and empirically validated strategies to optimize sustained engagement of attention in areas such as education, gaming, and therapy.

Recent studies are exploring the neural mechanisms supporting VR attention techniques by measuring eye movements during immersion. Quantifying gaze behavior offers empirical insights into the neurocognitive underpinnings shaping visual attention in action. Seo et al. [63] quantified visual attention using gaze patterns to demonstrate improved attentional processing toward avatar instructors, underscoring the importance of social presence for learning in VR. Moreover, Shavit-Cohen et al. [50] monitored gaze shifts between competing speakers to reveal auditory attention dynamics in multi-speaker VR environments. While significant progress elucidating attention’s role in VR has been made, open questions remain regarding predictive models and adaptable systems to dynamically optimize interfaces based on users’ differences and attention capabilities, motivating more personalized techniques grounded in cognitive and neural mechanisms.

## 3. Materials and Methods

In this section, we provide details on the experimental design and procedures used in this study. First, we introduce the outlier search game implemented in traditional (2D) and virtual reality environments. Next, we describe the study participants, apparatus, game structure, and trials. Finally, we outline the data collection process, pre-processing methods, feature extraction techniques, and classification models.

### 3.1. Outlier Search Game

The game was implemented in two environments: a traditional monitor, mouse, and keyboard setting versus an immersive VR system with a head-mounted display and hand controllers. The outlier search game requires players to identify anomalous images embedded within normal background images in 3D space within a time limit. Each trial had a time limit of 10 or 15 min. Each game shows a total of 260 images, of which 10 are outliers. In terms of their distribution, we used 5 different categories and showed players 50 normal and 2 outlier images per category.

One of our motivations for creating the outlier search game, or as the odd-one-out game is also called, was the popularity and simplicity of the game and the fact that it is often used in research to test various human cognitive abilities [66,67]. On the other hand, it has been shown that at a simple level, the recognition of outliers requires little to no knowledge from the participant, and an earlier implementation of the concept was tested in a domain expert collaboration task, but without the gamified context [68].

Before the actual data collection, we performed two pilot experiments to test the experimental setup and the game settings. The data recorded during this time was excluded from the evaluation. Based on the useful feedback, we clarified the instructions, refined the data recording protocol and limited the time and the number of outliers so that participants did not get too tired throughout the entire experiment (6 play sessions). Therefore, we set the maximum execution time to 15 min, the other version at 10 min and the number of outliers for each session at 10. Based on user experience feedback the time allowed and the number of tasks seemed appropriate for participants who were not familiar with VR games (they were all experienced with a traditional PC setup).

**Figure 2:**
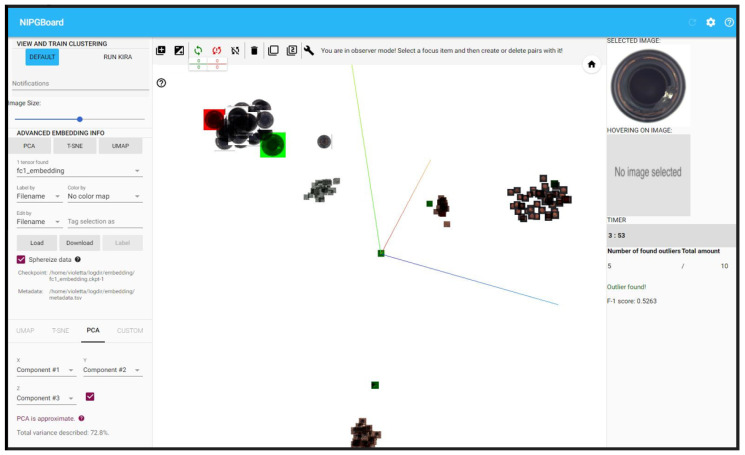
Example screenshot from the NipgBoard interface. On the (**left**), various display settings and dimension reduction options can be selected. Correctly selected items have a green overlay and incorrect selections have a red overlay, as shown in the (**upper left**) corner. In the middle, sample images from the MVTec Anomaly Detection dataset can be seen in the 3D projector panel after the PCA application. These grouped image sets represent the bottle, hazelnut, transistor, leather and tile categories. On the right side, the enlarged version of the currently selected image is presented, and below it, the timer, outlier counter, and F1 score are shown as text.

In the traditional setup, we used a custom plugin developed in Typescript and Python, incorporated into our NipgBoard tool—an interactive online system based on Google’s TensorBoard [69]. We used its embedding projector functionality to display the game in the Google Chrome web browser. A visualization of the NipgBoard’s interface can be seen in Figure 2. Participants were instructed not to alter interface settings or other functions.

**Figure 3:**
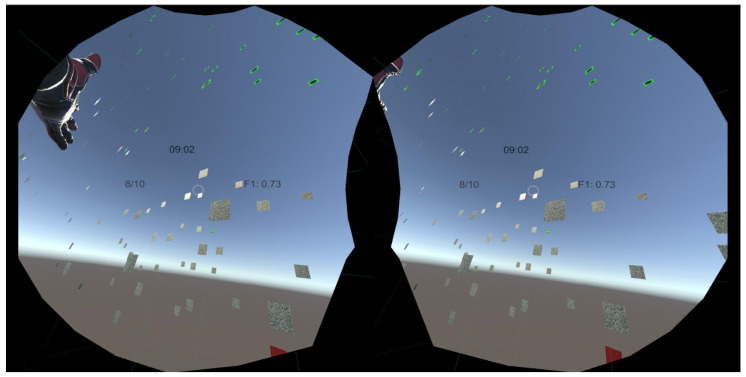
Screenshot from the HTC Vive Pro Eye headset’s VR view. In the (**upper left**) corner, the displayed gloves represent the player’s hand in the virtual environment, the small squares in the field of view are samples from the MVTec Anomaly Detection dataset. On the top, a green overlayed cluster of images (bottles) can be seen, as both outliers have been found there. In the (**bottom right**) corner, a red overlayed image (tile category) is an incorrect selection. From left to right, the displayed numbers in the middle are the outlier counter, the timer, and the current F1 score. The small transparent circle in the middle represents the target of the participant’s gaze.

The VR version was developed using Unity3D and C#, with the SteamVR plugin integrating the VR headset with the game. We utilized Unity Edition Professional, version 2020.3.1f1, as the game engine. The Unity MainCamera was replaced by the Player prefab camera. C# scripts enabled VR capabilities. An example view of the virtual reality environment is shown in Figure 3.

Graphical layouts utilized the MVTec Anomaly Detection image dataset [70]. This database contains 5000 high-resolution images in 15 classes, each with defective and defect-free examples. Images displaying scratches, cracks, contamination, or structural damage are labeled defective. Normal images exhibit no visible flaws. We selected the following 5 classes: bottle, hazelnut, leather, tile, and transistor with 50 normal and 2 anomalous samples each.

To enable 3D visualization of the image data, we first performed feature extraction using pre-trained deep neural networks (DNNs). For the traditional setting (2D), we used the VGG16 ImageNet model [71], while the VR version utilized ResNet50 [72]. We then applied dimensional reduction techniques on the extracted feature vectors to project the images into an embedded space. Principal component analysis (PCA) was used in both environments to reduce the features into three dimensions. For the VR game, we also applied t-distributed stochastic neighbor embedding (t-SNE) [73] for additional dimensionality reduction.

These methods produced well-separated, distinguishable image clusters based on the learned feature representations. Simple observation of the 3D visualizations showed a clear separation between the different image categories. The 2D and 3D environments were similar in terms of the display of the full image set: all 5 image clouds were in the field of view at launch and the participant had the freedom to choose which image set to observe first. The NipgBoard projection space allowed the participant to operate the zoom and rotation from an external view. In VR, the participant started from a central point and all the images surrounded his position, with navigation allowing him to bypass or even pass through the images.

**Figure 4:**
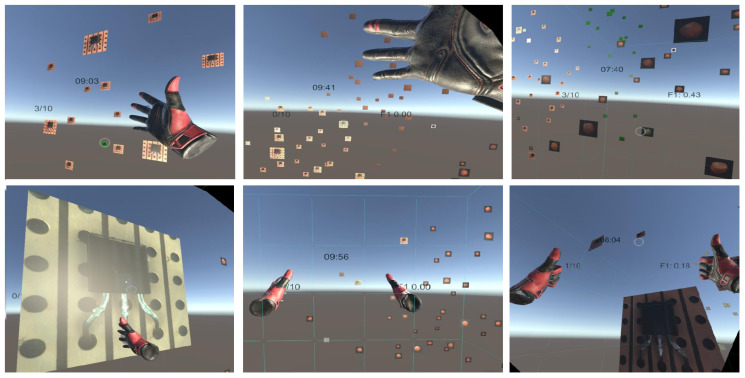
Illustrative screenshots from the outlier search game in the virtual reality environment. The (**top row**) shows the OHF mode, and the (**bottom row**) presents the THF technique. A blue grid appears due to proximity, alerting the person wearing the VR headset where the edges of the safe play area are in the real world. The screenshots show changes in ambient light conditions and instances where participants observe objects very close and far away.

To illustrate the visual possibilities offered by the virtual environment, Figure 4 shows a series of images captured from the VR view. To facilitate participants’ attention to the task, no extra visual background elements were used.

Differing user input modalities naturally characterize the traditional and VR scenarios. In the traditional setting, users navigate among the displayed images using a mouse or keyboard, with actions performed via mouse clicks. Conversely, in the VR environment, navigation is achieved through free movement in the designated play area, utilizing one or both controllers to execute interactions by pressing the controller buttons.

In general, we aimed to make the game environments as similar as possible in terms of task-solving, game mechanics and feedback methods. The biggest challenge was to create similar navigation in 2D and 3D spaces. The traditional input method in 2D is primarily the mouse, so since we wanted to simulate smooth and free mouse movement in the virtual environment, flying techniques seemed to be the appropriate method instead of jumping or teleporting in space.

It is a natural need that participants wish to receive real-time feedback on their performance. We wanted to avoid participants selecting too many images without actual observation or considered choice so that incorrect selections would be penalized. Their actual performance was reported back to them by the F1 value, the calculation of which was explained to them beforehand. To inform users about their real-time progress and the success or failure of their actions, various visual feedback mechanisms were implemented. Both game environments provided the following system responses:

- Green overlays on images following a correct selection;
- Red overlays on images after an incorrect selection;
- Blue overlays on images under active observation with the mouse cursor or gaze;
- After the successful selection of both outliers within an image group, all remaining images received green overlays;
- The remaining time, outlier counter, and F1 score were dynamically updated and displayed in the NipgBoard interface or in the VR field of view.

The NipgBoard interface further displayed feedback with text messages corresponding to correct, incorrect, and repeated selections. Moreover, to monitor user performance in real-time, we utilized commonly used evaluation metrics. The F1 score, an effective measure for classifier model efficiency, is a weighted average of precision and recall values, yielding a score between 0 (worst) and 1 (best). Precision measures the ratio of correct predictions to total predictions made by the model, while recall (true positive rate) represents the ratio of correct predictions to all possible correct predictions. In the context of the outlier search game, a correct prediction corresponds to a participant selecting a true outlier image, whereas an incorrect or false prediction arises when a normal, non-outlier image is selected.

To enable accurate calculations, deselecting images once they were selected was prohibited. The game concludes under two scenarios: either the time limit is reached, or all 10 outliers are found within the allocated time. Participant performance metrics were designated for incorrect image selection (misclicks), completion time, and F1 score for each game trial.

### 3.2. Experimental Design

**Figure 5:**
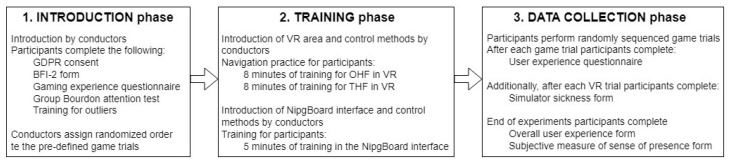
Flowchart of the experimental design with the three main phases: the introduction, training, and data collection. Each stage contains the list of official tests and questionnaires, the time schedule and the order of tasks for the conductors and participants.

For the sake of completeness, we describe the whole process of the experiments and data collection, but due to the focus of the current study on gaze-related analysis, the wider evaluation of the data collected from the questionnaires and other logs will not be detailed. The experimental design consisted of three main phases: introduction, training, and data collection. The flowchart with details of each stage is represented in Figure 5.

We started with an instruction phase, where the general concept of the experiment, outlier search game design, and different control methods of the game environments were explained to the participants. As part of this stage, the general data protection regulation (GDPR) form, the Big Five Inventory-2 (shortly BFI-2) personality traits test [29] and general information forms about prior gaming experience were filled out. Participants were also asked to complete the pen-and-paper Group Bourdon attention test. The game experience form was a short Likert-scale questionnaire—our original work.

To avoid any doubts about whether the image is an outlier or not, a short training session was also carried out in the introduction stage. During the training, two pairs per image class were presented, for a total of 10 comparison exercises. Participants had to compare the two given images and decide which one was the outlier. Questions and free discussion were allowed on the solutions.

The final step of this phase was to assign a random order to the six pre-defined game modes and to inform the participant accordingly. The possible variations of game settings in terms of environment, navigation technique, and time frame can be seen in Table 1.

In the training phase, participants were allowed to try out the following two navigation modes in virtual reality: one-handed flying (OHF) and two-handed flying (THF). Drawing on relevant literature, we chose navigation methods that are generally considered to be easy or enjoyable for users to learn but also sufficiently different to be comparable. According to the study of Drogemuller et al. [74] where they compared different, commonly used VR navigation techniques, they found that two-handed flying is the fastest and most preferred among the 25 involved participants. Based on their findings, one-handed flying was also reported as one of the methods that was easiest to understand and perform. On this basis, we decided to choose a rather simple method (OHF) and a more enjoyable but more complex one (THF). As they require the use of hands in different ways, they are sufficiently distinct to be further evaluated.

OHF is an unimanual navigation technique that indicates that the user is pointing in the desired direction, and the further the arm is stretched, the faster the movement. THF is a bimanual method for navigation in virtual environments, in which the user uses both hands to describe a vector that determines the direction and speed.

After the VR training, the traditional experiment setup was also introduced to the participants. The navigation can be performed with mouse movements and clicks or with the keyboard’s arrow keys. In the projection space of NipgBoard, one can zoom in/out using the mouse scroll wheel, shift position by holding down the right mouse button and moving the cursor, and rotate the camera view by holding down the left mouse button and moving the cursor. To practice the navigation in the traditional setting, we used a similar data set as in the experiment, and in the VR setting, we used the normal and defective elements of the following categories from the MVTec Anomaly database: carpet, grid, wood, and pill. The distribution of outliers and non-outliers was the same as in the actual experiment. In this phase, we did not record gameplay logs or personal data.

The number of outliers per game trial was the same for all participants, but to exclude the possibility that users remembered the location of the outliers, the defective samples were different in each attempt. We prepared six sets of data for the experiment and four backup sets.

The data collection phase was the final stage of the experimental work. In this session, participants were asked to solve the pre-selected, randomly sequenced outlier search tasks. The participants were tested individually. Each experiment took approximately three hours. Each participant had one attempt to complete the experimental stage. However, if dizziness or fatigue is reported while navigating in virtual reality, the experiment is stopped and the user can repeat it later with a different set of data. If any technical problems occurred, the game trial was also repeated. Participants were allowed extra rest time upon request. The outlier images were shown to the participants after each experiment as solutions to the gameplay.

Depending on the game parameters, the participants filled out questionnaires after game trials. After each game, participants responded to a Likert-scale questionnaire we created about their experiences in each game session. We aimed to collect the usability characteristics in terms of navigation, game environment, and time limit parameters.

Furthermore, if the environment was virtual reality, participants were asked to fill in a simulator sickness questionnaire (SSQ) [75] to indicate the subjective occurrence and severity of any symptoms they might have on a detailed symptom list. As the work of Bimberg et al. [76] shows, the SSQ can be applied to novel virtual reality research, despite its limitations.

After the six game trials had been completed, participants filled in a questionnaire on the overall user experience and a form measuring subjective sense of presence. The summary questionnaire was compiled by us and allowed the participants to compare and rate all game settings. The sense of presence form used was based on the work of Witmer et al. [77], where the relationship between task performance and the phenomenon of presence in a virtual environment was investigated. Lastly, in the form of a short, informal interview, participants were allowed to share their comments on the experiment.

In terms of data collection, in the traditional setting, we captured facial videos of the participants using a simple off-the-shelf HD webcam, gaze data with the Tobii Nano device, and mouse coordinates, screen capturing, keyboard, and game events using our custom python scripts. In the virtual reality environment, we collected the following data: user actions and game events, gameplay frames, navigation coordinates, and gaze data. For gaze data, we collected logs of gaze origin, gaze direction, and blinks, which were built-in variables in the HTC Vive Pro Eye.

#### Apparatus and Participants

Various virtual reality headsets are available in the market, and according to Angelov et al. [78], HTC Vive Pro is the best performer in terms of technical parameters. The final chosen apparatus for the experiment was the HTC Vive Pro Eye, which includes eye tracking. This device has a resolution of 1440 × 1600 per eye, a field-of-view of 110 nominal, and an optical frame rate of 90 Hz. To ensure eye tracking in the traditional game environment, we used the Tobii Pro Nano eye tracker attached below the monitor. This device has a sampling rate of 60 Hz, and it has a video-based eye tracking technique that relies on pupil and corneal reflection with dark and bright pupil illumination modes.

For running both game environments we used an AMD Ryzen 7 2700 eight-core computer, a 3.2 GHz processor with 32 GB RAM, an NVIDIA GeForce GTX-1080 video card, and a Windows 10 Home Operating System.

The actual experiments were carried out with the involvement of 30 participants, with 11 females and 19 males, aged from 20 to 41 (M = 26.26, SD = 4.37).

Most of the participants (29) were right-handed, and one person was ambidextrous. In terms of eyesight, 15 participants did not need vision correction, 2 wore contact lenses and 13 wore glasses. Regarding the experience with virtual reality, participants reported that 18 had no previous experience with VR, 5 had tried it occasionally but not in games, and 7 were familiar with VR games. Concerning experience in using the mouse, 18 participants declared themselves to be very skilled and used a gamer mouse, while 12 participants reported being sufficiently skilled in using a mouse.

The volunteers were instructed that data about their gameplays will be logged for further analysis and the answers from the additional tests and questionnaires will be used as well. The participants were asked to sign a GDPR consent form before the experiments, and the Ethics Committee of the Faculty of Informatics, Eötvös Loránd University approved the study. We anonymized personal data before further evaluation.

### 3.3. Collected and Derived Data as Ground Truth

This section provides details on the ground truth data collected and derived for the correlation and classification tasks. Separate subsections refer to game performance metrics from game logs, personality trait scores from questionnaires, and attention-related groups extracted from the Group Burdon test.

#### 3.3.1. Game Performance Metrics

To evaluate participant performance in each game session, we extracted two key performance metrics: misclicks and completion time. Misclicks refer to the number of erroneous clicks made on non-outlier images during each unique game session. A higher misclick count within a session implies diminished performance. Completion time represents the total time, measured in seconds, expended by a participant to complete each unique game session. Longer completion times within a given session indicate poorer performance.

These metrics were calculated based on game logs tracking correct selections, incorrect selections, and session duration. They provide quantitative measures of performance on the outlier search task. Moreover, a key relationship exists between completion time and misclicks, known as the speed-accuracy trade-off [79]. Specifically faster completion times often lead to more errors. By analyzing these metrics individually for each session, nuanced insights can be gained regarding how factors such as the environment, navigation method, and time constraints influence efficiency and accuracy over time.

##### Big Five Personality Traits

The Big Five Inventory-2 (BFI-2) questionnaire was administered to each participant to assess personality based on the five-factor model: extraversion, agreeableness, conscientiousness, neuroticism, and open-mindedness.

**Figure 6:**
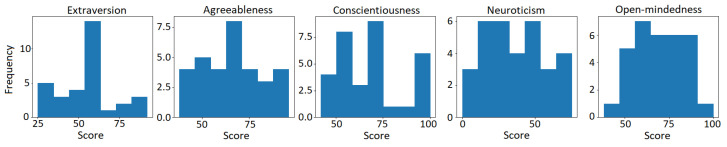
Score distributions for Big 5 Personality Traits calculated from the BFI-2 test, measured on a 5-point Likert scale. The values are scores between 0 and 100 for each trait.

The BFI-2 contains 60 items measured on a 5-point Likert scale. It generates a continuous score between 0 and 100 for each of the Big Five traits. These scores served as ground truth labels for training machine learning models to predict personality solely from gaze features. A visual overview can be seen in Figure 6.

#### 3.3.2. Attention Assessment Using the Group Bourdon Test

Simple psychological tests like the Bourdon–Wiersma dot cancellation test [8] and the Group Bourdon test, a variation in the previously mentioned attention test [7], are commonly used to measure attention ability and concentration levels in human studies. For example, Van der Linden et al. [9] used the Bourdon-Wiersma test to assess attention difficulties in individuals with professional burnout. Wolan et al. [80] applied the same test to measure improvements in children’s psychomotor development after dog-assisted therapy. The Group Bourdon test was used by Hoonhout et al. [81] to evaluate the effects of different lighting conditions on performance. These types of tests are brief, easy to administer online or on paper, and provide a straightforward analysis.

The Group Bourdon test [7] was, therefore, utilized in this study as a reliable means of evaluating participants’ attention abilities along the dimensions of processing speed and accuracy. This pen-and-paper test requires participants to visually scan structured point patterns on a sheet and identify target point groups while avoiding errors of commission and omission.

Specifically, each participant was instructed to mark only the groups of four points among configurations of three, four, or five points arranged in rows on a paper sheet. Five 1-min trials were administered, with participants scanning as far down the sheet as possible during each trial before the administrator stopped them. The last point group scanned was marked on each sheet to quantify progress. To obtain quantitative performance measures, the following raw metrics were manually counted after each trial:

- *N*: Total number of point groups scanned,
- $A_{c}$: Number of incorrectly marked groups (errors of commission),
- $A_{o}$: Number of missed target groups (errors of omission),
- $A_{e}=A_{c}+A_{o}$: Total number of errors,
- *t*: Total time (number of minutes).

Using these notations and definitions, two key performance indicators have been calculated to present the results of the Group Burdon test. The first is processing speed, which indicates an individual’s ability to detect, perceive and respond to rapid changes in the environment. In simple terms, it measures the speed of visual scanning and cognitive processing [82].

The second assessment is processing accuracy, which refers to the precision and quality of the selection. This measures the accuracy of an individual’s attention, which reflects the ability to perform tasks accurately under pressure [83].

Taken together, these measures provide a partial clinical picture of each participant’s attentional ability. We define the following attention-related groups as performance assessments of the Group Burdon test.

- Processing speed ($V_{p}$): total number of point groups scanned per unit time (Grp/minute)
  (Equation (1)),
- Processing Accuracy (*K*): ratio of correct responses to the total attempted answers
  (Equation (2)).

(1) $$V_{p}=\frac{N}{t}$$

(2) $$K=\frac{N-A_{e}}{N}$$

### 3.4. Data Pre-Processing

To enable categorization for the classification task, the continuous personality trait scores (ranging from 0 to 100) were divided into low, medium, and high categories using a data-driven binning approach. Specifically, boundaries for the three bins were determined by first inspecting the distribution of scores for each trait independently (see Figure 6). Thresholds were then defined at the 33% and 66% percentile scores, resulting in evenly spaced bins each containing approximately one-third of the participants. This binning strategy ensured a balanced classification while maximizing separation between categories based on the empirical score distributions.

**Figure 7:**
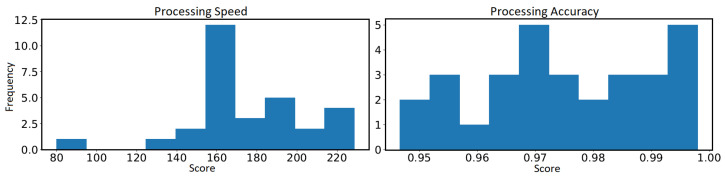
Calculation of performance metrics based on Group Burdon test scores. On the left, the score distribution can be seen for the processing speed (group/minute), which shows how many groups of points the participant observed during the given time. The right side of the graph shows the processing accuracy rate, which indicates the proportion of correct selections, taking into account both missed groups and incorrect selections.

Unlike personality traits, attention-related scores were discretized into two classes of high and low based on a similar data-driven binning approach. This binary representation was chosen for simplicity since no precedents existed for establishing standardized category boundaries. To determine the binning threshold, the distribution of processing speed values and processing accuracy ratios were analyzed (see Figure 7). The histograms revealed natural cut points that split participants into two evenly-populated groups—one with relatively high scores and one with lower scores. For processing speed, the threshold was identified at 170 groups/min. Values above this limit were labeled as high processing speed, while scores below it were designated as low processing speed. Likewise, for processing accuracy, the distribution suggested 0.975 as the cut point, with scores >0.975 categorized as high processing accuracy and scores <0.975 categorized as low processing accuracy.

While our initial discretization of personality traits into low, medium, and high categories was designed for the classification task, we adjusted our strategy for the correlation analysis. Specifically, to facilitate consistent statistical testing alongside the binary attention-related groups, we adopted an additional median split, consolidating the three personality trait categories into two classes (low and high).

This arbitrary split was solely employed to allow for an easier correlation analysis as well as to enable the use of uniform statistical methods. It is important to note that the correlation analysis is exploratory, and the presence of false positives (type I error) cannot be completely excluded, but the details of the statistical results and the effect size give a good indication of the results.

Finally, quality control checks revealed corrupted gaze log files for two participants. To enable analysis accuracy and data integrity, these individuals were excluded from all subsequent analyses.

### 3.5. Feature Extraction from Gaze Data

We pre-processed the raw gaze data via feature engineering. We first performed linear interpolation on the missing gaze vectors and then proceeded to extract eye movement events.

In the VR environment, we detected fixations and saccades using a modified Velocity-Threshold Identification (I-VT) suitable for the VR setting [84]. Since there is no prior knowledge on gaze velocity and duration thresholds for fixation and saccades detection in the VR setups. We first experimented with threshold values used in [62] but these yielded negative performance in the classification tasks. Therefore, we utilized I-VT in conjunction with the Median Absolute Deviation (MAD), which is a robust estimator of dispersion that is resilient to the influence of outliers and can automatically find a coherent separation threshold [85]. In the 2D environment, we utilized the Robust Eye Movement Detection for Natural Viewing (REMoDNaV) algorithm [86]. REMoDNaV is a robust eye movement detection method that accounts for variations in the distance between participants’ eyes and the eye tracker over time, making it suitable for scenarios with varying distances and ensuring its robust performance. Furthermore, it performs robustly on data with temporally varying noise levels.

During fixation, the visual gaze is sustained on a single location for a specific period. These fixations are valuable indicators of attention and cognitive processing activity [87,88]. As part of our features, we used fixation rate, which is the number of fixations per second, fixation duration, which is the total duration of fixations, and extracted three statistical descriptors, namely, mean, standard deviation, and max from the duration of the fixations.

Saccades provide valuable information and have been found to have a strong correlation with visual search behavior [89]. In the same manner as fixation, we used saccade rate, saccade duration, and the corresponding statistical descriptors from the duration.

To extract gaze features from the raw logs, we used CatEyes [90] Python Toolbox, which includes REMoDNaV and the modified I-VT. Initially, for each individual setting in 2D (2D-10, 2D-15) and VR (OHF-10, OHF-15, THF-10, THF-15), we extracted our set of fixation and saccades features. This resulted in a collection of 10 gaze features per setting. Then, we concatenated the features from all settings for each environment to form the final set of features, referred to as “2D-All” and “VR-All”.

Finally, to ensure the most effective predictive performance for both the Big Five traits and attention-related groups, we employed a feature selection approach to retain only the most informative gaze features. Firstly, we calculated the importance of each gaze feature using three different metrics, namely chi-squared, mutual information, and ANOVA F-value. Among these metrics, mutual information yielded the best results overall and was selected as our feature selection metric.

Then, we iteratively trained our models by increasing the number of features from k=1 to *T*, where *T* is the total number of features for each 2D/VR setting. Specifically, T=10 for individual settings in 2D/VR, T=20 for 2D-All, and T=40 for VR-All. We repeated this process until we obtained the best F1 average performance. This allowed us to identify the *k* gaze features with the highest mutual information scores and retain only those features in our final set of features. By doing so, we aimed to reduce the dimensionality of the feature space and improve the performance of our predictive models.

### 3.6. Machine Learning Classification Models

For the classification task, we evaluated the performance of four machine learning models including Random Forest (RF), K-Nearest Neighbors (KNN), Support Vector Machine (SVM), and Gradient Boost (GB):

- Random Forest (RF) [91] is a machine learning algorithm that uses an ensemble of decision trees to make predictions. This method involves constructing multiple decision trees on subsets of the training data and aggregating the results to obtain a final prediction. The hyper-parameters considered are the number of estimators and the depth.
- k-Nearest Neighbour (KNN) [92] is a non-parametric method based on the principle of finding the k-number of nearest neighbors to a given data point and making predictions based on the majority class or average value of these neighbors. The algorithm requires the choice of a distance metric and the selection of a value for k. Thus, only those two hyper-parameters were considered.
- Gradient Boost (GB) [93] is one of the most powerful tools for building predictive models for classification. It defines an ensemble prediction as a combination of weak learner models, which are typically decision trees. GB requires the selection of a learning rate, the number of estimators, and the maximum depth of trees, among others. Only those hyper-parameters were considered in our study.
- Support Vector Classifier (SVC) is a linear discriminative algorithm that seeks to find a hyperplane that separates the data into the respective classes in the case of classification. SVM requires the choice of a kernel function and a regularization parameter, C, that controls the trade-off between having a complex boundary and ensuring that the boundary does not over-fit the data. We only considered those hyper-parameters in our study.

### 3.7. Training Procedure

We employed a nested cross-validation strategy to train our models and fine-tune the selected hyperparameters. This involved using a 5-fold stratified cross-validation approach. For each iteration, the data were divided into a training set, validation set, and test set, with 20% of the participants being selected as the test set, 20% of the remaining participants as the validation set, and the rest as the training set. The data splits were performed in a participant-dependent manner to prevent overfitting and generalize the models to unseen data. The cross-validation was repeated 10 times to eliminate any participant-group effects on the model. Based on the validation results, the most optimized hyperparameters were selected using the F1 score as the main metric. The final model training was performed on the combined training and validation set and the performance evaluation using the held-out test set. The average F1 score and accuracy of a model are computed based on the results of all 50 iterations. Due to nearly balanced classes, the theoretical chance level was approximately 33% in the case of personality estimation and 50% in the case of attention-related group prediction.

## 4. Results

In this section, abbreviations will be utilized to refer to various navigation methods and given time limit combinations. For the VR settings, we use the following: OHF-10 (one-handed flying with a 10-min time limit), OHF-15 (one-handed flying with a 15-min time limit), THF-10 (two-handed flying with a 10-min time limit), THF-15 (two-handed flying with a 15-min time limit). For an extended evaluation, we also use VR-All, where all VR settings (all types of the aforementioned navigation method and time limit combinations) and features are concatenated. In terms of the 2D environment, the changing settings parameter was the time limit; thus, we use 2D-10 (10-min time limit) and 2D-15 (15-min time limit), as well as 2D-All, which is the concatenation of corresponding features of those two settings.

### 4.1. Correlation Results

Prior to the classification task, we conducted an investigation to explore the potential correlation between the two game performance metrics: the number of misclicks and completion time, which served as our dependent variables, and personality/attention-related groups, which were our independent variables. The purpose of this investigation was to determine whether there was a significant relationship between these variables and to provide a rationale for the importance of predicting these ground truths.

To examine such correlations, we employed a series of two-sample independent *t*-tests. To ensure that our *t*-tests were reliable, we only considered results that met the necessary assumptions for *t*-tests. Specifically, we assessed the normality of both our dependent and independent variables using the Shapiro–Wilk test and only included *t*-tests with values greater than 0.05, indicating that the variables were normally distributed. The independent *t*-tests revealed significant and marginally significant differences between our pairs of variables.

#### 4.1.1. Correlation between Game Performance and Attention-Related Groups

To compare performance between attention-related groups, we conducted *t*-tests on two sets of participants: those with high processing accuracy versus those with low processing accuracy, and those with high processing speed versus those with low processing speed. These comparisons were based on the performance metrics described previously in Section 3.4 and visualized in Figure 7.

The results did not reveal any significant differences in the 2D environment. Therefore, only the results from the VR will be reported. Table 2 summarizes the results of the relationship between attention-related groups and performance in VR tasks.

In the processing accuracy groups, for the OHF-10 setting, participants with lower processing accuracy levels completed the task significantly faster compared to those with higher processing accuracy levels.

For the processing speed groups, for the THF-10 setting, participants with higher processing speed levels demonstrated significantly more misclicks and longer completion times compared to the participants with lower processing speed levels. The OHF-15 and THF-15 settings also show marginally significant associations, with participants with higher processing speed levels exhibiting more misclicks and longer completion times compared to participants with lower processing speed levels.

#### 4.1.2. Correlation between Game Performance and Personality Traits

To examine performance differences among personality groups, we conducted *t*-tests for each of the Big Five traits. Particularly, we compared participants with high levels of a trait against those with low levels of the same trait. The basis for these comparisons was the performance metrics outlined earlier in Section 3.4.

Table 3 summarizes the results exploring the relationship between personality traits and performance within 2D and VR environments. It outlines specific task settings, the personality traits investigated (of which yielded positive results—extraversion, conscientiousness, open-mindedness), and dependent variables of interest (misclicks and completion time). Mean values (with standard deviations) are provided for high and low groups within each trait. Statistical results (t-values and *p*-values) reveal the significance of these correlations, while Cohen’s d demonstrates the effect size of each personality trait on task performance.

In the 2D environment, regarding extraversion, participants in the high extraversion group demonstrated significantly more misclicks and longer completion times within the 2D-10 setting. Interestingly, the 2D-15 setting revealed that participants in the high Conscientiousness group showed significantly shorter completion times and marginally fewer misclicks. Finally, higher levels of open-mindedness in the 2D-10 setting were marginally associated with longer completion times, but interestingly, were also linked to significantly fewer misclicks.

Within the VR environment, participants in the high conscientiousness group (OHF-15 setting) exhibited significantly shorter completion times. Those in the high agreeableness group showed significantly longer completion times (OHF-15 setting) and significantly more misclicks (OHF-10 setting). Conversely, participants in the high extraversion group demonstrated significantly more misclicks in the OHF-10 setting and marginally more misclicks within the THF-15 setting.

### 4.2. Classification Results

Table 4 and Table 5 show a performance comparison of our four models (RF, KNN, GB, and SVC) for the prediction of the Big Five traits (refer to Table 4) and attention-related groups (refer to Table 5) in both VR and 2D environments. In the VR environment, the evaluation was performed using four individual settings (OHF-10, OHF-15, THF-10, THF-15) and the corresponding feature concatenation (VR-All). The evaluation in the 2D setting was performed using two individual settings (2D-10, 2D-15) and the corresponding feature concatenation (2D-All). We report the average F1 score and performance accuracy for each model across every setting and ground truth.

Firstly, in terms of personality prediction (see Table 4), we evaluated the concatenation of features (VR-All) in both settings. In the VR environment, KNN demonstrated the strongest performance for agreeableness (F1 = 43.32, acc = 44.73), conscientiousness (F1 = 52.21, acc = 52.02), and neuroticism (F1 = 46.07, acc = 47.35), while GB exhibited higher performance for extraversion (F1 = 54.77, acc = 53.33). For open-mindedness, SVC performed well (F1 = 41.62, acc = 40.05), and RF performed above the theoretical chance level but did not surpass the performance of any other models.

In the 2D environment, RF exhibited the strongest performance for agreeableness (F1 = 39.15, acc = 38.45). KNN achieved the best performance for neuroticism (F1 = 49.77, acc = 50.01), extraversion (F1 = 48.94, acc = 46.01), and open-mindedness (F1 = 39.01, acc = 42.64). Conversely, no model outperformed theoretical chance levels for conscientiousness.

In both the VR and 2D settings, the highest-performing model in terms of F1 score generally also demonstrated the highest accuracy, which may be attributed to the balanced ground truth. However, we observed exceptions, including open-mindedness in 2D, as well as conscientiousness and extraversion in VR settings.

Next, we analyzed the performance of individual settings. We found that for each combination of trait and setting, at least one of the models used in this study performed above theoretical chance levels in predicting personality traits. Notable exceptions were conscientiousness in 2D-10 and open-mindedness in 2D-15, where no model achieved above-chance performance. This indicates that personality trait prediction is generally a viable task, regardless of the specific setting. Additionally, the optimal model varied across traits and settings. However, KNN consistently outperformed other models for conscientiousness in VR and neuroticism in 2D.

Moreover, our investigation of attention-related groups in Table 5 revealed a distinct performance within VR-All and 2D-All settings. Regarding processing speed, the SVC model achieved the highest performance in the VR-All setting (F1 = 76.81, acc = 76.00). In 2D-All, GB and KNN exhibited comparable superiority (KNN: F1 = 61.51, acc = 61.50; GB: F1 = 61.02, acc = 62.40). For processing accuracy across both 2D-All and VR-All settings, RF consistently outperformed other models (VR-All: F1 = 79.02, acc = 79.00; 2D-All: F1 = 72.03, acc = 73.89).

To delve deeper into individual settings, as previously noted in personality prediction, the highest-performing model is dependent on the ground truth predicted and the specific setting used. Within the VR environment, a clear divide emerges for processing speed: RF dominates in OHF-10 (F1 = 69.87, acc = 69.67) and OHF-15 (F1 = 58.15, acc = 58.33), while KNN excels in THF-10 (F1 = 78.00, acc = 78.00 ) and THF-15 (F1 = 68.05, acc = 68.33). For processing accuracy, KNN consistently leads in THF-10 (F1 = 67.34, acc = 69.70), OHF-15 (F1 = 52.01, acc = 56.00), and THF-15 (F1 = 57.15, acc = 60.75). Interestingly, OHF-10 sees comparable performance between KNN (F1 = 78.06, acc = 78.30) and RF (F1 = 78.81, acc = 79.00). The 2D environment presents different results: RF demonstrates superiority in processing speed across 2D-10 (F1 = 72.52, acc = 73.40) and 2D-15 (F1 = 55.88, acc = 59.10). RF also maintains the highest performance in processing accuracy for 2D-10 (F1 = 71.92, acc = 73.00), but SVC performs best in 2D-15 (F1 = 63.81, acc = 69.69). GB did not surpass the performance of any other model.

Table 6 and Table 7 represent the average F1 score trait-wise and setting-wise, with only the best-performing models taken into account. We adopted two averaging approaches: trait-wise and setting-wise. For the former, we calculated the F1 score by averaging across all settings for each Big Five trait or attention-related group (processing speed and processing accuracy). For the latter, we averaged the F1 score across all Big Five traits or attention-related groups (processing speed and processing accuracy) for each setting. We conducted this analysis for both the 2D and VR environments, with Table 6 representing F1 averages setting-wise for personality prediction and attention-related groups and Table 7 representing F1 averages trait-wise.

Regarding personality prediction for trait-wise average performance (Table 7), we observed a clear distinction in the most accurately classified traits between VR and 2D environments. Neuroticism exhibited the highest classification performance in 2D, while extraversion led in VR. Conversely, agreeableness consistently demonstrated the lowest classification performance across both environments. Additionally, in VR, agreeableness and open-mindedness displayed similar performance levels. Moreover, our analysis highlights significant disparities in classification performance between the two environments. Specifically, conscientiousness and extraversion yielded higher prediction performance in VR compared to 2D. In contrast, neuroticism and open-mindedness exhibited superior performance in the 2D setting compared to VR.

Regarding setting-wise comparisons (Table 6), our analysis revealed notable findings. In VR, feature fusion within VR-All outperformed individual VR settings, demonstrating the benefits of data aggregation. Interestingly, 2D-All performance was comparable to that of 2D-10. Additionally, task duration significantly impacted personality prediction performance. Settings with a 10-min duration yielded superior results across both VR and 2D environments compared to those with a 15-min duration. Furthermore, two-handed VR settings proved highly effective for personality estimation compared to the one-handed setting.

Regarding attention group prediction, Table 7 reveals a distinct performance contrast between VR and 2D. While 2D environments excelled in predicting overall processing accuracy, VR settings demonstrated superior performance for predicting processing speed. When considering setting-wise comparisons (Table 6), a notable pattern emerges: feature fusion across all settings (2D-All, VR-All) consistently outperformed in both environments. Additionally, within VR, the 10-min task setting consistently yielded the highest performance.

Interestingly, mirroring our findings in personality trait prediction, the type of hand control significantly impacted performance in attention-related prediction with two-hand VR settings being the most effective and the 10-min setting being optimal for both VR and 2D environments.

## 5. Discussion

In this section, we first examine the key correlations found between game performance metrics (completion time, number of misclicks) and the measured human factors like attention-related and personality traits. We then overview the classification results for predicting processing speed, processing accuracy and personality from gaze, including model comparisons and performance differences across traits and settings. Finally, we analyze the overall classification results through average performance measures.

### 5.1. Correlation Findings for the Attention-Related Groups

We compared participants’ game performance across 2D and VR environments, focusing on their attention-related group (high proc. accuracy vs. low proc. accuracy and high proc. speed vs. low proc. speed). Interestingly, significant differences were observed only in the VR environment, with no noteworthy findings in the 2D environment. This may result from the immersive nature of VR, known to elicit more engaging and realistic responses from participants [94].

The findings indicate that differences between high and low processing accuracy groups become more evident in the one-handed flying setting with a 10-min time limit. This suggests that the performance gaps between processing accuracy groups become more pronounced when participants are constrained to using one hand and have a shorter time frame to complete the task. In other task settings, including two-handed flying and extended time limits, these distinctions are less apparent.

When comparing the 10-min task settings (OHF-10 and THF-10) to the 15-min task settings (OHF-15 and THF-15), we found that a shorter time limit resulted in more pronounced differences between processing speed groups. In the 10-min settings, participants in the higher processing speed groups exhibited an increased number of misclicks and longer completion times. On the other hand, the 15-min settings revealed less distinct differences between the processing speed groups, with only marginal variations observed in completion times and misclicks. This suggests that time constraints may affect the prominence of differences between processing speed groups, with tighter time limits potentially emphasizing the disparities between high and low processing speed participants. In accordance with the findings in [95], under time pressure, attention narrows, leading to decreased efficiency in perceptual processing and ultimately resulting in reduced performance.

The trade-off between processing accuracy and processing speed is a well-established phenomenon in cognitive psychology, often referred to as the speed-accuracy trade-off [79]. By utilizing the Group Burdon test as a dependable metric to measure processing speed and accuracy, our findings suggest that categorizing individuals according to their attention test outcomes allows us to effectively discern their performance in the outlier search game within the VR setting.

### 5.2. Correlation Findings for Personality Traits

For a more comprehensive interpretation, the evaluation was also applied to compare groups of game performance metrics and personality traits in both game environments.

In the 2D environment, participants with high extraversion scores displayed a higher number of misclicks across both VR and 2D environments. Longer game completion times were observed as well; however, this finding was limited to the 2D environment. In alignment with [58], no significant difference was found concerning task completion time in the VR environment. These observations align with existing literature, suggesting that individuals with high levels of extraversion are more likely to engage in risk-taking behavior and demonstrate a greater risk propensity [96,97,98]. Extraverted individuals might perform poorly under monotonous conditions or in vigilance tasks, as they may not be as involved in the task as introverts and are more likely to commit fatigue-related errors [99].

We observed notable performance differences related to conscientiousness levels in both 2D and VR environments. Participants with low conscientiousness demonstrated significantly longer completion times in the 15-min settings, while a slightly higher number of misclicks were observed solely in the 2D environment. The relationship between conscientiousness and task-solving performance in our study is supported by [100,101], who emphasized the tendency of conscientious individuals to be more organized, disciplined, and achievement-oriented. Although our findings regarding the number of misclicks are marginal, they align with studies on conscientiousness and accident involvement [102,103,104], indicating that conscientious individuals generally make fewer errors across various settings.

In the 2D environment, a slight association was observed between high open-minded-ness and longer completion times in the 10-min setting. Participants with high open-mindedness also demonstrated a significantly lower number of misclicks in the same setting. Our results imply that open-mindedness might have a subtle impact on performance in 2D tasks, while no significant differences were observed in the VR environment.

The findings suggest that individuals who score high on open-mindedness may be more attentive and cautious, which could lead to fewer errors. However, their exploratory nature might lead to longer completion times, as they engage more thoroughly with the task at hand. As reported in [105] open-mindedness is positively related to cognitive ability and intellectual engagement. The relationship between open-mindedness and performance in our study can be attributed to the cognitive and behavioral tendencies associated with this trait. Consequently, this relationship led to more careful decision-making and a reduced error rate, albeit with slightly longer completion times.

Regarding agreeableness, our study did not yield any significant results in the 2D environment. However, in the VR environment, participants with lower levels of agreeableness completed the task in a significantly shorter time, while those with higher levels of agreeableness demonstrated a greater number of misclicks. The literature on the relationship between agreeableness and individual task performance presents varied and inconclusive results. Consistent with our findings, individuals with higher levels of agreeableness tend to make more errors in assigned tasks. Empirical research supports this, showing that individuals with higher conscientiousness outperform their more agreeable counterparts in self-regulation tasks [106]. Regarding the number of misclicks in our study, our results align with [107], which found that individuals with higher agreeableness may display an optimistic tendency, leading to faster decision-making when clicking on objects, even if those objects are incorrect.

### 5.3. Classification Results

Our personality classification results (see Table 4) highlight the potential of gaze features in predicting personality traits in both 2D and VR environments. Our results demonstrate above-chance-level (33%) predictions in most settings, regardless of the task settings we controlled for, such as time limits in 2D and both time limits and hand control in VR, with performance not exceeding chance levels for conscientiousness (2D-10 and 2D-All) and open-mindedness (2D-15). To the best of our knowledge, our study is the first to explore personality trait predictions from gaze in a VR environment, building on previous laboratory-based studies that established a link between personality traits and eye movement characteristics [13,20,108,109]. The results show that optimal models vary across traits and settings, suggesting that a one-size-fits-all approach may not be suitable for all scenarios. It is important to note that the 3D stimulus in our 2D and VR environments is challenging, requiring different cognitive factors we did not account for in this study, such as cognitive load and memory capacity. Future research could investigate these cognitive factors.

Regarding attention-related group prediction, our findings show that RF was the top-performing model in both 2D-All and VR-All for predicting processing accuracy only, in contrast to personality results where no single model outperformed the rest across both 2D-All and VR-All. KNN ranked second in certain individual settings, likely due to its strength in handling a smaller number of features. The classification results for attention-related groups suggest gaze features may have the potential for predicting processing accuracy and processing speed levels as categorized by the Group Bourdon test, with performance surpassing chance levels. Considering the associations found in our correlation results between game performance and attention-related groups, we suggest that predicting attention-related groups holds significant potential for enhancing smart interface design. For instance, as suggested by previous studies on user-adaptive systems [110,111], adaptive interfaces can be developed by considering an individual’s attention-related attributes such as processing speed and processing accuracy levels.

For individuals with low processing accuracy, the interface could be more explicit, asking users to confirm their decisions, while for higher processing speed individuals, the pace of the interface could be slowed down to ensure fewer mistakes are made. It should also be noted that a person’s cognitive abilities may vary significantly depending on other environmental factors such as fatigue, motivation, cognitive load, tolerance of monotony, etc. Incorporating assistive tools that interact with users based on their attention-related levels could further enhance their experience and performance [112]. These insights may prove valuable in practical applications, such as making hiring decisions or customizing interfaces, where assessing individuals based on specific traits is crucial.

### 5.4. Performance Average

In Table 6 and Table 7, we examined the effects of time constraints and hand control types on the classification performance of personality traits and attention-related groups in both 2D and VR environments. We found that the predictability of personality traits varied between the two environments.

In 2D, neuroticism was the most accurately predicted trait, with performance surpassing 50%, while the remaining traits had performance within the 39–45% range. On the other hand, consciousness and extraversion showed higher predictability in VR than in 2D. This variation can be attributed to factors such as immersion, user interaction, and sensory feedback, which influence the expression of personality in VR environments. Studies have shown that the increased sense of presence in VR enables users to express their personality traits more naturally, as they engage in highly immersive experiences [94,113]. This is in contrast to the limited keyboard and mouse inputs available in 2D environments. Moreover, multi-sensory feedback in VR [114] fosters realistic and emotionally engaging experiences, potentially eliciting more pronounced behaviors linked to specific personality traits.

The reasons for neuroticism’s higher predictability in 2D remain unclear. One possible explanation is the game-induced frustration levels experienced by participants, which may be captured and measured through the neuroticism trait. In 2D settings, repetitive and less varied interactions may inadvertently reveal patterns associated with neuroticism, such as higher levels of anxiety, stress, or frustration. Furthermore, performance on tasks in 2D environments may be influenced by emotional states closely related to neuroticism, such as difficulty concentrating [115].

In the case of attention-related group prediction, when comparing 2D and VR environments, we observed that 2D interfaces are more favorable for processing accuracy in attention-related tasks, which could be explained by their simpler, more familiar layout that reduces cognitive load, allowing for a more focused and precise attentional behavior. On the other hand, VR environments excel in boosting attention’s processing speed, and a possible explanation could be attributed once again to the immersive and engaging nature of VR that stimulates quicker, more reflexive responses as well as more real-life engagement. This immersive aspect, by creating a sense of presence and urgency, may activate faster cognitive processing as users navigate through the more complex and dynamic VR space.

When analyzing the effects of time constraints and hand control types, we observed that two-handed controls in VR setups were, on average, the most effective for personality prediction. Additionally, a 10-min task duration yielded superior results. We believe that two-handed controls provide a more natural and intuitive interaction within the virtual environment, enabling users to perform a wider range of actions and gestures [116]. This increased level of interaction can result in a more immersive experience, thereby enhancing the expression of users’ personalities. The performance discrepancy in personality prediction between the 10-min and 15-min VR settings could stem from factors such as task engagement, fatigue, and learning effects [117,118]. Participants in a 10-min setting may remain more engaged and focused during the shorter task duration, leading to clearer and more consistent behavioral patterns that facilitate personality prediction [119].

Regarding attention-related groups, time constraints, and hand control types, we found that tasks lasting 10 min and using one-handed control demonstrated superior performance, aligning with the observation that shorter durations often lead to better prediction in personality predictions. Interestingly, the performance difference between 10- and 15-min tasks was less pronounced when employing two-handed controls. In comparing one-handed versus two-handed controls, regardless of task duration, a significant performance advantage was noted for the two-handed control options.

Our analysis did not show any advantages of fusing gaze features in 2D personality prediction, as better results were obtained in the 10-min settings compared to 2D-All. The fusion of features from our 2D sessions might have introduced noise or redundancy, negatively affecting model performance. In contrast, merging features from VR sessions resulted in a modest yet significant improvement in performance. For attention-related groups (processing accuracy and processing speed), our results show that combining different features from individual settings works well in both 2D and VR environments, leading to better attention-related group prediction performance, unlike in personality trait prediction. Our findings also suggest that feature concatenation across various settings can enhance performance, particularly in VR, encouraging future research to explore more sophisticated feature fusion methods.

## 6. Limitations and Future Work

The results and conclusions of our study are primarily limited to the specific context of gaze behavior in relation to personality traits and attention-related groups during our VR/2D task. The impact of these factors on various types of tasks involving different navigation and locomotion techniques or more complex tasks remains uncertain. Moreover, our findings may not necessarily generalize to different settings or populations. Therefore, we perceive this work as a starting point for further exploration of the relationship between personality traits, attention-related groups, and gaze behavior within various interaction techniques and types of tasks, both within and beyond the VR/2D environments.

The relatively small sample size of 28 participants could impact our results, potentially leading to a higher variance in our models’ performance and limiting the generalizability of our findings. Despite this limitation, we managed to achieve above-baseline performance. Furthermore, our study focused primarily on the predictability of personality traits and attention-related groups, without investigating the individual contributions of saccade and fixation features. Moreover, our study utilized a minimal subset of gaze features, excluding velocity-based features and smooth pursuit. Incorporating a more comprehensive set of gaze features in future work could provide deeper insights into the relationship between gaze behavior and the psychological aspects they represent.

Another set of limitations in our study relates to potential confounding factors that may influence our statistical results. For instance, we did not account for the possibility of a learning effect, whereby participants may improve their performance over time due to repeated exposure to the VR/2D tasks. Additionally, the possible impact of fatigue on participants’ performance may not have been adequately addressed in the study design. Prolonged periods of task engagement can lead to mental and physical fatigue, which can harm gaze behavior and other cognitive processes. Lastly, one potential confounding variable in our statistical study is the order in which participants completed the experiments (2D and VR). Because we did not counterbalance the order of the experiments, some participants may have completed the VR experiment first, while others completed the 2D experiment first. This order effect could introduce systematic biases in our correlation results.

Finally, since the statistical analysis is exploratory and aims to provide a rationale for the classification task, we did not apply strict corrections for multiple comparisons, which may lead to an increased chance of false positive findings (Type I errors).

## 7. Conclusions

Our results represent a novel approach to exploring the relationship between gaze behavior, personality traits and attentional features in both 2D and VR environments using machine learning techniques.

In our experiments, we have taken advantage of the immersive virtual environment and latent eye tracking beyond the traditional gaming environment to obtain data on personality traits during more natural behaviors, which is a more convenient method and a better experience than personality tests. Additionally, our study stands out for introducing the concept of attention-related characteristics (processing accuracy and processing speed) as ground truth and their relationship with participants’ game performance.

We developed multiple classification models based on the extracted gaze features. Several models exhibited superior performance, achieving above-chance performance levels in all settings. We found detectable correlations between performance in the outlier search game and participants’ attention-related groups and certain personality traits. The immersive nature of VR and its increased sense of presence appear to influence the expression of specific traits, such as extraversion and conscientiousness, leading to more pronounced gaze behavior patterns that facilitate prediction.

Based on the obtained results, we believe that time constraints and types of manual control may affect the classification performance of personality traits and attention-related groups. Furthermore, we observed differences in classification performance between VR and 2D environments, highlighting the potential impact of the environment on gaze behavior and prediction accuracy.

Ultimately, our findings have practical implications for the development of adaptive interfaces and assistive tools that cater to individual users’ attention levels and personality traits. By leveraging gaze behavior to predict psychological aspects, we can design more personalized and effective user experiences in various domains, ranging from education to entertainment.

## Figures and Tables

**Table 1:** List of the six pre-defined game trials. The variables are the game environment, navigation methods, and given time intervals.

Game Environment	Navigation Method	Time Interval
Virtual Reality	One-handed Flying (OHF)	10 min
Virtual Reality	One-handed Flying (OHF)	15 min
Virtual Reality	Two-handed Flying (THF)	10 min
Virtual Reality	Two-handed Flying (THF)	15 min
Traditional setting	Mouse and Keyboard	10 min
Traditional setting	Mouse and Keyboard	15 min

**Table 2:** Results for Group Burdon performance metrics and VR game performance comparison. It outlines task settings, attention-related groups (high vs. low), and dependent variables (misclicks and completion time). Mean values with standard deviations are provided for both high and low categories for either processing accuracy or processing speed. Statistical testing results (t-values and *p*-values) indicate the significance of differences between groups, while Cohen’s d measures the effect size of the attention mechanism on performance. Note: Negative Cohen’s d values indicate that the mean of the “Low” group is higher than the mean of the “High” group, reflecting the direction of the effect size.

Task Setting	Attention-Related Group	Dependent Variable	Mean (High) ± SD	Mean (Low) ± SD	t-Value	*p*-Value	Effect Size (Cohen’s d)
VR (OHF-10)	Proc. Accuracy (ratio)	Completion time (s)	582.91 ± 56.05	458.29 ± 146.69	46.0	0.004	1.122
VR (OHF-15)	Proc. Speed (group/min)	Misclicks (counts)	3.53 ± 3.1	1.6 ± 1.82	154.0	0.054	0.759
VR (OHF-15)	Proc. Speed (group/min)	Completion time (s)	682.02 ± 199.44	515.29 ± 241.75	151.0	0.056	0.752
VR (THF-10)	Proc. Speed (group/min)	Misclicks (counts)	3.47 ± 2.75	1.4 ± 1.14	171.5	0.015	0.983
VR (THF-10)	Proc. Speed (group/min)	Completion time (s)	695.13 ± 238.42	508.34 ± 234.49	161.0	0.04	0.79
VR (THF-15)	Proc. Speed (group/min)	Completion time (s)	563.7 ± 118.39	439.22 ± 216.24	144.5	0.069	0.714

**Table 3:** Correlation results for personality traits and dependent variables in both 2D and 3D game settings. Statistical testing results (t-values and *p*-values) indicate the significance of differences between groups, while Cohen’s d measures the effect size of the personality trait on performance. Note: negative Cohen’s d values indicate that the mean of the “Low” group is higher than the mean of the “High” group, reflecting the direction of the effect size.

Task Setting	Personality Trait	Dependent Variable	Mean (High) ± SD	Mean (Low) ± SD	t-Value	*p*-Value	Effect Size (Cohen’s d)
2D (10 min)	Extraversion	Misclicks (counts)	2.07 ± 1.84	0.73 ± 0.85	165.5	0.02	0.935
2D (10 min)	Extraversion	Completion time (s)	505.27 ± 105.07	375.13 ± 164.85	164.0	0.019	0.941
2D (15 min)	Conscientiousness	Completion time (s)	417.27 ± 226.79	603.74 ± 228.32	−52.0	0.046	−0.819
2D (15 min)	Conscientiousness	Misclicks (counts)	1.18 ± 2.25	3.63 ± 3.73	−41.0	0.066	−0.795
2D (10 min)	Open-mindedness	Completion time (s)	495.15 ± 124.25	398.18 ± 159.09	148.0	0.09	0.679
2D (10 min)	Open-mindedness	Misclicks (counts)	1.08 ± 1.14	4.0 ± 4.07	57.0	0.02	0.977
VR (OHF-15)	Conscientiousness	Completion time (s)	453.39 ± 186.55	682.76 ± 221.54	−46.0	0.009	−1.120
VR (OHF-10)	Extraversion	Misclicks (counts)	2.87 ± 2.36	1.07 ± 1.53	178.0	0.024	0.905
VR (OHF-15)	Agreeableness	Completion time (s)	675.55 ± 220.88	483.31 ± 211.65	−152.0	0.029	−0.889
VR (OHF-10)	Agreeableness	Misclicks (counts)	2.61 ± 2.41	1.0 ± 1.29	159.0	0.049	0.833
VR (THF-15)	Extraversion	Misclicks (counts)	3.2 ± 3.06	1.53 ± 1.96	154.5	0.09	0.650

**Table 4:** Comparative performance for personality traits across 2D and VR settings. The models’ F1 score and accuracy are displayed. The highest score is indicated by the values in bold.

Trait	Model	VR Setting										2D Setting					
		OHF-10		THF-10		OHF-15		THF-15		VR-All		2D-10		2D-15		2D-All	
		F1	acc.	F1	acc.	F1	acc.	F1	acc.	F1	acc.	F1	acc.	F1	acc.	F1	acc.
Agreeableness	GB	31.28	29.35	34.41	32.05	**39.59**	**37.34**	38.57	38.79	35.74	37.34	34.02	32.24	**39.63**	**40.22**	33.19	35.25
	KNN	35.25	34.05	**37.02**	**37.35**	37.77	36.72	**49.31**	**50.03**	**43.32**	**44.73**	**40.32**	**39.01**	36.42	39.82	33.42	34.62
	RF	**38.45**	**37.36**	30.12	28.01	37.45	35.33	36.15	36.73	34.72	34.73	30.74	33.03	36.56	37.87	**39.15**	**38.45**
	SVC	33.01	32.03	33.23	32.72	30.65	30.01	39.79	40.75	35.19	35.31	33.55	32.21	32.75	33.24	35.05	36.65
Conscientiousness	GB	35.73	38.01	46.72	48.03	46.15	48.05	39.84	41.37	49.38	50.07	**33.38**	**34.04**	30.41	29.88	28.84	29.23
	KNN	**47.41**	**49.36**	**49.27**	**50.72**	**49.76**	**52.01**	**42.15**	42.72	**52.21**	52.02	32.02	32.24	**37.18**	**38.01**	**33.47**	**33.06**
	RF	37.79	41.34	49.23	50.71	45.42	48.07	28.94	31.36	50.27	**52.77**	29.39	32.22	30.22	31.45	28.87	29.45
	SVC	39.59	48.01	41.33	47.33	38.42	40.71	41.17	**44.06**	42.15	41.36	31.63	31.04	28.47	28.62	31.74	32.44
Extraversion	GB	**37.68**	**37.34**	51.64	50.75	**42.86**	**42.72**	**57.79**	**57.32**	**54.77**	53.33	27.09	27.89	33.86	36.26	31.15	32.22
	KNN	35.71	36.73	43.52	44.74	39.13	36.76	46.69	47.37	48.17	45.39	35.17	37.27	**45.32**	**44.44**	**48.94**	**46.01**
	RF	31.75	34.00	**54.12**	**54.75**	34.57	36.79	42.96	45.35	53.36	**54.76**	28.45	28.88	40.76	40.85	30.23	29.25
	SVC	30.77	29.35	51.33	51.39	38.35	40.76	45.54	46.73	49.51	49.35	**42.86**	**44.00**	22.56	25.41	35.28	35.46
Neuroticism	GB	31.67	32.76	44.17	45.39	29.76	29.35	36.85	38.04	44.11	45.35	34.85	35.25	41.42	43.88	38.32	49.41
	KNN	37.14	40.02	**48.48**	**50.01**	**34.70**	35.36	**38.79**	43.32	**46.07**	**47.35**	**51.74**	51.84	**51.14**	**57.26**	**49.77**	50.01
	RF	36.15	41.34	43.43	44.04	34.55	39.34	32.04	34.03	43.94	44.77	37.02	46.22	44.11	45.47	40.43	41.24
	SVC	**40.93**	**46.01**	32.64	44.74	34.24	**45.35**	31.84	**46.07**	32.45	44.02	41.17	**52.65**	41.47	40.25	41.51	**52.64**
Open-mindedness	GB	**50.04**	**50.06**	30.83	32.07	38.89	37.32	27.26	26.07	38.19	38.07	45.67	45.81	28.92	31.24	38.85	39.64
	KNN	45.56	46.05	**41.42**	**40.78**	**49.47**	**47.35**	**40.25**	**40.05**	38.89	38.01	**49.16**	**49.47**	**33.91**	36.88	**39.01**	**42.64**
	RF	40.39	40.01	26.16	28.02	39.16	38.73	24.62	24.04	35.72	34.72	39.23	41.67	28.22	31.82	37.25	39.68
	SVC	31.59	34.03	39.45	38.70	35.12	36.05	35.08	36.00	**41.62**	**40.05**	40.26	43.42	29.93	**37.03**	37.88	41.66

**Table 5:** Group Burdon prediction performance (attention-related categories) for 2D and VR settings. The models’ F1 score and accuracy are displayed. The highest score is indicated by the values in bold.

Attention-Related Group	Model	VR Setting										2D Setting					
		OHF-10		THF-10		OHF-15		THF-15		VR-All		2D-10		2D-15		2D-All	
		F1	Acc.	F1	Acc.	F1	Acc.	F1	Acc.	F1	Acc.	F1	Acc.	F1	Acc.	F1	Acc.
Processing Speed	GB	69.23	69.00	74.89	74.67	53.70	54.00	64.53	64.33	68.75	69.00	68.91	69.80	44.93	47.10	61.02	**62.40**
	KNN	68.65	68.33	**78.00**	**78.00**	57.66	**58.33**	**68.05**	**68.33**	68.87	68.61	64.96	64.20	55.73	59.90	**61.51**	61.50
	RF	**69.87**	**69.67**	73.58	73.33	**58.15**	**58.33**	63.44	63.33	72.00	71.33	**72.52**	**73.40**	**55.88**	59.10	59.86	61.20
	SVC	51.01	51.33	67.66	67.33	56.77	57.33	60.40	60.67	**76.81**	**76.00**	53.72	60.90	48.30	**60.90**	47.65	56.60
Processing Accuracy	GB	73.44	73.00	64.84	66.00	51.74	52.70	45.31	46.38	71.24	71.36	70.50	71.00	59.94	61.68	67.52	68.90
	KNN	78.06	78.30	**67.34**	**69.70**	**52.01**	56.00	**57.15**	**60.75**	75.95	76.79	**73.83**	**74.70**	62.00	63.36	66.24	67.91
	RF	**78.81**	**79.00**	56.77	59.77	50.62	53.00	50.76	53.39	**79.02**	**79.00**	71.92	73.00	62.25	64.00	**72.03**	**73.89**
	SVC	69.34	69.72	57.37	61.00	45.00	**58.00**	48.73	52.74	67.02	68.00	61.88	66.77	**63.81**	**69.69**	63.89	65.23

**Table 6:** Setting-wise presentation of F1 scores averaged over personality traits and attention-related groups.

Category	VR Setting					2D Setting		
	VR-All	THF-10	THF-15	OHF-10	OHF-15	2D-10	2D-All	2D-15
Personality	47.55	46.04	45.59	43.22	42.84	43.45	43.45	41.39
Attention	77.93	72.66	72.59	64.34	55.08	66.77	73.15	61.31

**Table 7:** A comprehensive look at F1 score averages over settings, presented trait-wise for personality traits and group-wise for attention-related groups.

Category	Traits	VR	2D
Personality	Agreeableness	41.48	39.65
	Conscientiousness	48.12	41.67
	Extraversion	49.39	41.61
	Neuroticism	44.51	50.82
	Open-mindedness	41.74	45.66
Attention-related	Proc. Accuracy	66.86	70.85
	Proc. Speed	70.18	63.30

## Data Availability

Data is contained within the article.
